# COVID-19 Mortality and Vaccine Coverage — Hong Kong Special Administrative Region, China, January 6, 2022–March 21, 2022

**DOI:** 10.15585/mmwr.mm7115e1

**Published:** 2022-04-15

**Authors:** Dallas J. Smith, Avi J. Hakim, Gabriel M. Leung, Wenbo Xu, W. William Schluter, Ryan T. Novak, Barbara Marston, Bradley S. Hersh

**Affiliations:** ^1^CDC COVID-19 Response International Task Force, Atlanta; ^2^Epidemic Intelligence Service, CDC; ^3^Li Ka Shing Faculty of Medicine, University of Hong Kong, Hong Kong Special Administrative Region, China; ^4^National Institute for Viral Disease Control and Prevention, Chinese Center for Disease Control and Prevention, Beijing, China; ^5^CDC China, Beijing, China.

On January 6, 2022, a cluster of COVID-19 cases* caused by the Omicron variant of SARS-CoV-2, the virus that causes COVID-19, was detected in Hong Kong Special Administrative Region, China (Hong Kong), resulting in the territory’s fifth wave of COVID-19 cases (*1*). This wave peaked on March 4, 2022, with 8,764 COVID-19 cases per million population (*2*), resulting in a total of 1,049,959 cases and 5,906 COVID-19–associated deaths reported to the Hong Kong Department of Health during January 6–March 21, 2022.^†^ Throughout this period, the COVID-19 mortality rate in Hong Kong (37.7 per million population) was among the highest reported worldwide since the COVID-19 pandemic began (*3*). Publicly available data on age-specific vaccination coverage in Hong Kong with a 2-dose primary vaccination series (with either Sinovac-CoronaVac [Sinovac], an inactivated COVID-19 viral vaccine, recommended for persons aged ≥3 years or BNT162b2 [Pfizer-BioNTech], an mRNA vaccine, for persons aged ≥5 years), as of December 23, 2021,^§^^,^^¶^ and COVID-19 mortality during January 6–March 21, 2022, were analyzed. By December 23, 2021, 67% of vaccine-eligible persons in Hong Kong had received ≥1 dose of a COVID-19 vaccine, 64% had received ≥2 doses, and 5% had received a booster dose. Among persons aged ≥60 years, these proportions were 52%, 49%, and 7%, respectively. Among those aged ≥60 years, vaccination coverage declined with age: 48% of persons aged 70–79 years had received ≥1 dose, 45% received ≥2 doses, and 7% had received a booster, and among those aged ≥80 years, 20%, 18%, and 2% had received ≥1 dose, ≥2 doses, and a booster dose, respectively. Among 5,906 COVID-19 deaths reported, 5,655 (96%) occurred in persons aged ≥60 years**; among these decedents, 3,970 (70%) were unvaccinated, 18% (1,023) had received 1 vaccine dose, and 12% (662) had received ≥2 doses. The overall rates of COVID-19–associated mortality among persons aged ≥60 years who were unvaccinated, who had received 1 COVID-19 vaccine dose, and who had received ≥2 vaccine doses were 10,076, 1,099, and 473 per million population, respectively; the risk for COVID-19–associated death among unvaccinated persons was 21.3 times that among recipients of 2–3 doses in this age group. The high overall mortality rate during the ongoing 2022 Hong Kong Omicron COVID-19 outbreak is being driven by deaths among unvaccinated persons aged ≥60 years. Efforts to identify and address gaps in age-specific vaccination coverage can help prevent high mortality from COVID-19, especially among persons aged ≥60 years.

The Chinese Center for Disease Control and Prevention and the U.S. CDC conducted a descriptive analysis of COVID-19 incidence, mortality, age-specific vaccination coverage, and booster dose coverage after introduction of the Omicron variant in Hong Kong.^††^ Relative risks were calculated using mortality rates (deaths per million persons) by vaccination status and age, with the referent groups being ≥2-dose recipients; persons aged <30 years; or, within specific age groups, receipt of ≥2 vaccine doses. Data were obtained from publicly available sources, primarily the Hong Kong Department of Health (*2*) and Our World in Data (*3*). This activity was reviewed by CDC and was conducted consistent with applicable federal law and CDC policy.^§§^

During February 2020–December 2021, Hong Kong reported 12,649 COVID-19 cases and 213 associated deaths. On January 6, 2022, the first cluster of COVID-19 cases attributable to the Omicron variant were identified in guests in a hotel for compulsory quarantine after arrival in Hong Kong from abroad (*1*). Daily COVID-19 incidence increased sharply, from 1.7 per million population on January 6 to a peak of 8,764.2 per million on March 4, before declining to 2,716.0 by March 21, 2022. By February 14, 2022, 100% of sequenced isolates were Omicron variant, BA.2 lineage.

As of December 23, 2021, two thirds (67%) of vaccine-eligible persons overall in Hong Kong had received ≥1 COVID-19 vaccine dose, 64% had received ≥2 doses, and 5% had received a booster dose (Table 1). Vaccination coverage varied by age; among persons aged 30–59 years, 82%, 80%, and 5% had received ≥1 dose, ≥2 doses, and a booster dose, respectively. Among persons aged ≥60 years, approximately one half (52% and 49%) had received ≥1 and ≥2 vaccine doses, respectively, and 7% had received a booster dose. Coverage declined with increasing age: 48% of persons aged 70–79 years and 20% of those aged ≥80 years had received ≥1 vaccine dose, 45% and 18% had received ≥2 doses, and 7% and 2% had received a booster dose.

**TABLE 1:** COVID-19 vaccination coverage, by age group — Hong Kong Special Administrative Region, China, December 23, 2021

Age group, yrs	No. of doses received/vaccination coverage*		
	≥1 dose no./total no. (%)	≥2 doses no./total no. (%)	Booster^†^ no./total no. (%)
3–29	980,945/1,784,800 (55)	869,096/1,784,800 (49)	14,471/1,784,800 (0.8)
3–19	345,393/976,100 (35)	255,510/976,100 (26)	730/976,100 (0.1)
20–29	635,552/808,700 (79)	613,586/808,700 (76)	13,741/808,700 (2.0)
30–59	2,817,846/3,443,000 (82)	2,751,916/3,443,000 (80)	171,899/3,443,000 (5.0)
30–39	889,354/1,126,300 (79)	864,294/1,126,300 (77)	32,943/1,126,300 (3.0)
40–49	983,239/1,142,500 (86)	963,035/1,142,500 (84)	63,356/1,142,500 (6.0)
50–59	945,253/1,174,200 (81)	924,587/1,174,200 (79)	75,600/1,174,200 (6.0)
≥60	1,049,110/2,034,100 (52)	1,004,606/2,034,100 (49)	145,989/2,034,100 (7.0)
60–69	701,148/1,071,800 (65)	679,592/1,071,800 (63)	96,451/1,071,800 (9.0)
70–79	266,706/560,500 (48)	253,378/560,500 (45)	39,761/560,500 (7.0)
≥80	81,256/401,800 (20)	71,635/401,800 (18)	9,777/401,800 (2.0)
**Total**	**4,847,901/7,261,900 (67)**	**4,625,618/7,261,900 (64)**	**332,359/7,261,900 (5.0)**

**Source:** COVID-19 Vaccination Programme. https://www.covidvaccine.gov.hk

* Total persons vaccinated divided by total population in the age group.

^†^ In Hong Kong, booster doses are considered third and fourth doses after the 2-dose primary COVID-19 vaccination series vaccines.

A total of 5,906 COVID-19–related deaths were reported in Hong Kong during January 6–March 21, 2022 (Table 2). The daily mortality rate increased from zero on January 6 to 34.8 per million on March 21 and peaked at 37.7 on March 14. Among all deaths, 4,118 (70%) occurred in unvaccinated persons and 5,655 (96%) occurred in persons aged ≥60 years. Unvaccinated decedents aged ≥60 years (3,970) accounted for 67% of total deaths, and among the 5,655 deaths in persons aged ≥60 years, 70% were in unvaccinated persons. Unvaccinated decedents aged ≥70 years (3,661) and ≥80 years (3,036) accounted for 62% and 51% of all deaths, respectively.

**TABLE 2:** COVID-19–associated mortality,* by age group and vaccination status — Hong Kong Special Administrative Region, China, January 6–March 21, 2022

**Age group, yrs**	**Total no. of deaths^†^ (% of total)**	Age-specific mortality*	**No. of deaths, by no. of vaccine doses**			Mortality,* by no. of vaccine doses		
			**None**	**1**	**≥2**	**None**	**1**	**≥2**
**Total**	**5,906 (100)**	**799**	**4,118**	**1,068**	**720**	**4,277**	**317**	**129**
<30	21 (0.4)	11	13	4	4	29	6	4
<3	1 (0.0)	8	1	0	0	8	0	0
3–11	5 (0.1)	9	3	2	0	13	8	0
12–19	5 (0.1)	11	3	1	1	158	7	3
20–29	10 (0.2)	12	6	1	3	92	4	4
30–59	228 (4.0)	66	133	41	54	1,039	23	17
30–39	15 (0.3)	13	8	3	4	140	6	4
40–49	43 (0.7)	38	30	4	9	1,000	6	8
50–59	170 (2.9)	145	95	34	41	2,317	52	39
≥60	5,655 (95.9)	2,780	3,970	1,023	662	10,076	1,099	473
60–69	496 (8.4)	463	309	94	93	2,784	168	108
70–79	977 (16.5)	1,743	625	201	151	5,841	786	396
≥70	5,159 (87.4)	5,363	3,661	929	569	12,936	2,490	1,061
≥80	4,182 (70.8)	10,408	3,036	728	418	17,250	6,207	2,696

* Deaths per million population.

^†^ Age was unknown for two unvaccinated decedents.

Overall, the relative risk of dying from COVID-19 among unvaccinated persons in Hong Kong was 33.2 times the risk among persons who received ≥2 doses (Table 3). Compared with persons aged <30 years, mortality risk among those aged ≥60 years was 252.7 times as high, and among persons aged ≥80 years was 946.2 times as high. Among persons aged ≥60 years, the relative risks for death among those who were unvaccinated were 21.3 times the risk among persons who had received ≥2 doses and 2.3 times the risk among those who had received 1 vaccine dose.

**TABLE 3:** COVID-19 mortality* and relative mortality risk^†^ among persons aged <30 years, 30–59 years, and ≥60 years, overall and by age and vaccination status — Hong Kong Special Administrative Region, China, January 6–March 21, 2022

Characteristic	Mortality rate*	Relative mortality risk^†^
**Overall no. of COVID-19 vaccine doses received**		
≥2	129	Ref
1	317	2.5
0	4,277	33.2
**All vaccination groups, by age group, yrs**		
<30	11	Ref
30–59	66	6
≥60	2,780	252.7
60–69	463	42.1
70–79	1,743	158.5
≥80	10,408	946.2
**No. of doses received, by age group, yrs**		
**<30**		
≥2	4	Ref
1	6	1.5
0	29	7.3
**30–59**		
≥2	17	Ref
1	23	1.4
0	1,039	61.1
**≥60**		
≥2	473	Ref
1	1,099	2.3
0	10,076	21.3
**60–69**		
≥2	108	Ref
1	168	1.6
0	2,784	25.8
**70–79**		
≥2	396	Ref
1	786	2.0
0	5,841	14.7
**≥80**		
≥2	2,696	Ref
1	6,207	2.3
0	17,250	6.4

**Abbreviation:** Ref = referent group.

* Deaths per million population.

^†^ Compared with referent group of ≥2 doses.

## Discussion

After the emergence of the Omicron variant in Hong Kong in early January 2022, COVID-19 cases increased rapidly, resulting in 5,906 deaths as of March 21, 2022. At the start of this outbreak, immunity in Hong Kong was presumed to be predominantly vaccine-derived as a result of a dynamic COVID-Zero strategy, whereby after successful containment, every case is investigated, and measures are implemented to interrupt onward transmission (*4*). Although overall 2-dose vaccination coverage was 64%, rates varied between age groups and were lower among older adults: 2-dose vaccination coverage was 63% among persons aged 60–69 years, 45% among those aged 70–79 years, and 18% among those aged ≥80 years. New Zealand, a country with a much lower population density than Hong Kong, also had largely vaccine-derived immunity. Although New Zealand’s 2-dose COVID-19 vaccination coverage was 95% among persons aged ≥60 years, the country experienced a similar increase in incidence after introduction of Omicron; however, mortality in New Zealand peaked at 2.1 per million population per day compared with 38.0 in Hong Kong (*5*). These findings align with data from existing studies indicating that the risk for death from COVID-19 increases with age and reinforce the effectiveness of vaccination in preventing death from the Omicron variant in older adults (*6*,*7*).

COVID-19 vaccine-induced immunity wanes over time, but booster vaccinations can elicit a strong immune response and restore vaccine effectiveness (*7*). At the beginning of the Omicron wave in Hong Kong, only 7% of persons aged ≥60 years had received a booster dose, including just 2% of those aged ≥80 years. The primary series of COVID-19 vaccines plus a booster dose is more effective at preventing severe outcomes caused by the Omicron variant than a primary series alone (*8*). In addition to the low vaccination coverage among persons aged ≥60 years, waning immunity since the last vaccine dose could have contributed to COVID-19–associated mortality in Hong Kong.

The reasons for low COVID-19 vaccination coverage among older persons in Hong Kong are not clear. Low vaccine confidence has presented major hurdles for governments aiming to reduce COVID-19 transmission and mortality. A June 2021 survey in Hong Kong found that 56.8% of participants were hesitant about or resistant to receiving a COVID-19 vaccine (*9*). The dynamic COVID-Zero strategy, successful until the emergence of the Omicron variant, might have resulted in further complacency, particularly among older persons. A survey conducted during November 2020–January 2021 in China found that older adults were more likely to accept a COVID-19 vaccine if they perceived themselves to be at high risk for infection or had trust in the government (*10*). Experience with the COVID-19 pandemic can motivate public health officials to increase vaccine distribution and coverage. Hong Kong targeted older persons for vaccination during the outbreak. As of March 21, 2022, 2-dose COVID-19 vaccination coverage in Hong Kong has increased substantially, to 81% among persons aged 60–69 years, 69% among persons aged 70–79 years, and 39% among persons aged ≥80 years (*3*).

The findings in this report are subject to at least four limitations. First, summary-level data were analyzed, and other risk factors for death, including comorbidities, could not be examined. Second, completeness of reporting of COVID-19–attributed deaths is unknown. Third, immunity due to previous infection could not be assessed; however, such immunity was likely low given that few cases had been reported during previous waves (*4*). Finally, vaccine effectiveness can vary by type and timing of vaccination, which were not accounted for in this analysis.

During January–March 2022, data from Hong Kong suggested that higher mortality rates were driven by low vaccination coverage among older adults. These data underscore the importance of monitoring age-specific vaccination coverage and implementing strategies that increase COVID-19 vaccination coverage among all population groups, especially those most at risk for severe illness. Efforts to identify disparities in age-specific vaccination rates and address gaps in vaccination coverage among groups at high risk can help prevent high mortality from COVID-19, especially in older adults.

**Summary**

**What is already known about this topic?**

COVID-19 vaccines are important tools to protect populations from severe disease and death.

**What is added by this report?**

Among persons aged ≥60 years in Hong Kong, 49% had received ≥2 doses of a COVID-19 vaccine, and vaccination coverage declined with age. During January–March 2022, reported COVID-19–associated deaths rose rapidly in Hong Kong. Among these deaths, 96% occurred in persons aged ≥60 years; within this age group, the risk for death was 20 times lower among those who were fully vaccinated compared with those who were unvaccinated.

**What are the implications for public health practice?**

Efforts to identify and address gaps in age-specific vaccination coverage can help prevent high mortality from COVID-19, especially in older adults.

## Footnotes

* A hotel cluster of COVID-19 cases on January 6, 2022, is thought to have been the origin of the fifth wave of cases, based on genomic surveillance data from sequences uploaded to GISAID. Before January 6, previous Omicron cases with different sequences were detected from sporadic introduction and community transmission (https://www.ceo.gov.hk/eng/pdf/article20220128.pdf); 50 cases were detected as of December 28, 2021 (https://www.ceo.gov.hk/eng/pdf/article20211228.pdf).

† Daily new confirmed COVID-19 cases and deaths per million persons listed are 7-day rolling averages.

§ Vaccination rates and vaccine-derived immunity were calculated 14 days before the introduction of the Omicron variants leading to Hong Kong’s fifth wave.

¶ Sinovac is recommended in persons aged ≥3 years. For persons aged ≥18 years, a 28-day interval between the first and second dose, a 28-day interval for immunocompromised persons, and a 90-day interval for the general population (priority for those aged ≥60 years) between the second and third dose is recommended; a fourth dose is recommended 90 days after the third dose for immunocompromised persons. Pfizer BioNTech vaccine is recommended for persons aged ≥5 years. For persons aged ≥18 years, a 56-day interval is recommended between the first and second doses, a 28-day interval for those who are immunocompromised, and a 90-day interval for the general population (priority to those aged ≥60 years) between the second and third dose; a fourth dose with an interval of 90 days after the third dose is recommended for immunocompromised persons. https://www.covidvaccine.gov.hk/en/vaccine

** Age was unknown for two unvaccinated decedents.

†† Death counts were obtained from the Hong Kong Department of Health, which provides the most up-to-date mortality data, but these data might differ slightly from other sources because of differences in completeness. The government of Hong Kong has established processes for linking case and vaccination data. COVID-19–associated death is defined as a death in a person who received a positive SARS-CoV-2 test result who died within 28 days of the collection date of the first positive specimen. The underlying cause of death might have been unrelated to COVID-19.

§§ 45 C.F.R. part 46.102(l)(2), 21 C.F.R. part 56; 42 U.S.C. Sect. 241(d); 5 U.S.C. Sect. 552a; 44 U.S.C. Sect. 3501 et seq.
